# Colorectal carcinoma: nucleosomes, carcinoembryonic antigen and ca 19-9 as apoptotic markers; a comparative study

**DOI:** 10.1186/1423-0127-18-50

**Published:** 2011-07-25

**Authors:** Jehad M Al-Shuneigat, Samir S Mahgoub, Fazlul Huq

**Affiliations:** 1 Faculty of Medicine, Department of Pharmacology and Biochemistry Mu'tah University, Al Karak, Jordan; 2 Faculty of Medicine, Discipline of Biomedical Science, The University of Sydney, Australia

**Keywords:** colorectal carcinoma, nucleosomes, carcinoembryonic antigen (CEA) and CA 19-9, tumor marker

## Abstract

**Background:**

Colorectal carcinoma is a common and often fatal disease in which methods of early detection and monitoring are essential. The present study was conducted for measuring serum levels of nucleosomes, carcinoembryonic antigen (CEA) and CA 19-9 in patients newly diagnosed with colorectal carcinoma and confirmed by clinicopathological study.

**Method:**

Thirty subjects were included in the current study: six normal subjects as a control group with mean age (45.6 ± 7.9) and twenty four colorectal carcinoma patients with mean age (46.9 ± 15.6), which were classified pathologically according to the degree of malignant cell differentiation into well differentiated (group I), moderately differentiated (group II) and poorly differentiated (group III). Fasting venous blood samples were collected preoperative.

**Results:**

The results revealed a significant increase in serum level of nucleosomes in patients with poorly differentiated tumors versus patients with well differentiated tumors (p = 0.041). The levels of CEA and CA19-9 showed no significant increase (p = 0.569 and 0.450, respectively).

**Conclusion:**

In conclusion, serum level of nucleosomes provides a highly sensitive and specific apoptotic marker for colorectal carcinoma.

## Background

Colorectal carcinoma is one of the leading causes of cancer-related death [1,2]. The 5-year survival rate for colon tumours in Europe ranges from 26% to 56% for men and from 29% to 59% for women. These differences in survival have been attributed to the stage and timing of diagnosis and, in some regions, to the quality of medical care [3]. Hence the need for early detection methods in colorectal cancer [4].

Tumor markers are used clinically for diagnosis, staging, and monitoring of the disease. They are proteins released from dying tumor cells or produced by neoplastic cells. There are two subcategories of these proteins, specific and non-specific. The specific proteins are expressed only in the tumor cells and are very useful for the detection and diagnosis of specific malignant tumors. Non-specific proteins or markers related to malignant cells are oncofetal or carcinogenic antigens, such as carcinoembryonic antigen (CEA), alphafetoprotein (AFP), prostate specific antigen (PSA), carbohydrate antigens CA15.3 and CA19-9. Recently nucleosomes, cytokeratine 18, and cyto-c in serum have been examined as markers for the evaluation of apoptotic death [5].

The basic unit of chromatin, known as a nucleosome, is composed of local wrapping of a short stretch of double stranded (ds) DNA (147 bp in length) around an octameric histone protein core of two molecules each of histones H_2_A, H_2_B, H_3 _and H_4 _(known as nucleosome core particle (NCP)), and the so-called linker DNA that varies between 10 and 100 bp and connects neighboring nucleosomes in a chain like pattern [6].

Apoptosis or programmed cell death has evolved in multicellular organisms to remodel tissue during development. It maintains tissue homeostasis (proliferation and apoptosis balance) by removing senescent cells and deleting cells with irreparable genetic damage. It is a highly regulated process with distinct morphologic and biochemical features [7,8].

The mechanism of apoptosis in tumors is unclear. However, it is known to be p53 and caspases dependent [9], a hallmark of cancer cells [10]. During apoptosis, caspases are activated leading to degradation of cell constituents [11]. High levels of circulating mono- and oligo-nucleosomal fragments are expected. In blood, circulating nucleic acids are not digested by endonucleases due to their close association with histone protein. High levels of circulating DNA fragments have been found in the blood of patients with most types of malignancy including colorectal, lung, gastrointestinal, breast, gynecological, renal, and nasopharyngeal cancers as well as lymphoma [12,13].

For colorectal cancer many biomarkers have been assessed but only a small number have been recommended for clinical use. The aim of the present study was to compare the circulating levels of nucleosomes, CEA and CA 19-9 as apoptotic markers of colorectal carcinoma and to determine which one is more specific and sensitive for clinical use.

CEA is a product of columnar and goblet cells in the normal colon cells as well as colonic cancer cells with a half life of 3-11 days. It is a glycoprotein with a molecular weight of 200 kDa; most of its carbohydrate content is composed of mannose, galactose, N-acetylglucosamine, fructose and sialic acid [14]. The serum levels of CEA may increase 4.5 to 8 months before the development of cancer symptoms. Therefore, CEA monitoring is the most cost-effective indicator for the disease [15].

CA 19-9 is an antigen originally isolated for the first time from human colorectal carcinoma, which is identified by monoclonal antibody designated 19-9. It was postulated that CA 19-9 could not be recommended for early diagnosis of colorectal carcinoma and its serial determination appears to provide little information to that of CEA in monitoring patients [16].

## Methods

The present study was conducted in patients with colorectal cancer admitted to the Gastroenterology Department of EI-Minia University Hospital. Twenty four patients were included in the study (ten females and fourteen males) with ages ranging from 34 to 72 years (mean 46.9 ± 15.6). Six normal subjects were selected as a control group. All patients proved to have colorectal carcinoma by history, examination, investigations and biopsy. The blood samples were collected before surgical interference.

The circulating levels of nucleosomes were estimated by cell death detection ELISA (CDDE)^plus ^supplied from Roche Diagnostic-Germany. CEA and CA 19-9 serum levels were determined by electrochemi-luminescence immunoassay (ECLISA) on Roche Elecsys 1010 immunoassay analyzer. The kits were used according to the manufacturer's instructions. The chemicals were supplied by Roche Diagnostics GmbH, D 68298, Mannheim, Germany. The patients were classified into three groups, nine patients with well differentiated tumor, nine patients with moderately differentiated tumor and six patients with poorly differentiated tumor according to The Modified Dukes classification of Astler and Coller [17].

### Statistical methods

To test for normal distribution, frequency of data was plotted against normal distribution curve. Nonparametric statistical methods were used. Frequency, median, range and standard error of means were used to describe data. Kruskal-Wallis, nonparametric analysis of variance was used to test for variability between groups in quantitative variables while, Mann-Whitney u test was used to test for significance of difference in quantitative variables between each two groups. Nonparametric Kendall's correlation was used to test for linear relationship between different quantitative variables. Regression analysis was used for further analysis of the linear relationship between nucleosomes, CEA and CA 19-9. Receiver operating characteristic (ROC) curve analysis was done using MedCalc software for evolution of sensitivity and specificity of different markers. P value was considered significant if less than 0.05. These tests were run on an IBM compatible personal computer using the Statistical Package for Social Scientists (SPSS) for windows 7.5 (SPSS Inc., Chicago, IL, USA).

## Results

Table (1) shows range, median and SE of the studied parameters in control versus patient groups (well, moderately and poorly differentiated tumors). There is significant increase in nucleosomes levels in all groups versus the control group (P < 0.001). CEA levels show a significant increase in all groups except the poorly differentiated tumor group versus the control group (P < 0.001 for the well differentiated tumor group, 0.037 for the moderately differentiated tumor group and 0.106 for the poorly differentiated tumor group). There is no significant increase in the levels of nucleosomes, also, no significant decrease in the levels of CEA with decreased grade of tumor differentiation. There is significant increase in the levels of nucleosomes in poor differentiated versus well differentiated tumors (p = 0.041).

**Table 1:** Comparison between serum levels of nucleosomes, CEA and CA 19-9 in the three patient groups versus the control group and each group of patients versus the other groups

Parameters Groups			Nucleosomes (AU)	CEA (ng/ml)	CA 19-9 U/ml
Control group (n = 6)	Median		282.6	2.3	20.9
	
	S.E ±		26.4	0.39	3.2
	
	Range	Min.	181.5	0.8	7.8
		
		Max.	519.2	4.2	41.4

Group I Patients (well differentiated tumor) (n = 9)	Median		1081	6.6	32.86
	
	S.E ±		209.5	9.00	101.6
	
	Range	Min.	712.4	1.29	8.4
		
		Max.	3382.5	1729	1895

Group II Patients	Median		1776.2	4.61	23.9
	
(moderate differentiated tumor) (n = 9)	S.E ±		171.3	75.6	64.6
	
	Range	Min.	772.2	1.6	1.6
		
		Max.	3426.9	2400	2246

Group III	Median		1860	4.6	34
	
Patients (poor differentiated tumor) (n = 6)	S.E ±		260.6	6.26	44.66
	
	Range	Min.	1149.7	1.72	3.16
		
		Max.	3482.5	52.6	386.2

P control versus group I			<0.001	<0.001	0961

P control versus group II			<0.001	0.037	0.666

P control versus group III			<0.001	0.106	0.816

P group I versus group II			0.086	0.464	0.116

P group I versus group III			0.041	0.569	0.450

P group II versus group III			0.566	0.861	0.627

**Figure 1:**
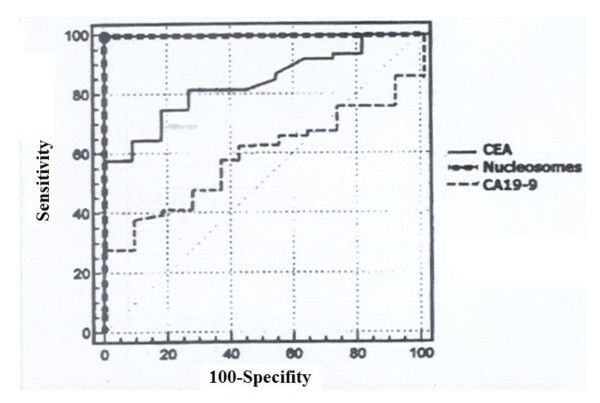
**Receiver operating characteristic curve (ROC) of nucleosomes, CEA and CA 19-9 in colorectal carcinoma diagnosis**.

The overall positive rates obtained from ROC curve (Figure 1) of circulating CEA and CA 19-9 (using the cutoff values of 3.56 ng/ml and 28 U/ml, respectively) were 56.2% and 36.4% (Table 2).

**Table 2:** Statistical evaluation of nucleosomes compared to CEA and CA 19-9 for colorectal carcinoma

Parameter	Cutoff value	Sensitivity %	Specificity %	J index	Accuracy %	Kappa		PPV %	NPV %	ROC curve area
						**Value**	**P**			

CA 19-9	28 u/ml	36.4	88.9	26	41	0.121	0.088	91.56	21.62	0.524

CEA	3.56 ng/ml	56.2	100	55.8	62.2	0.290	<0.001	100	30.40	0.808

Nucleosomes	421	100	100	100	100	1.00	<0.001	100	100	1.0

Table (2) shows statistical evaluation of nucleosomes in comparison to CEA and CA 19-9 for colorectal carcinoma. By all statistical parameters, nucleosomes show the best values. As regards kappa it shows an excellent agreement between laboratory and actual diagnosis for nucleosomes while the values of CEA and CA 19 -9 show only weak agreement.

The curve shows the true positive rate (sensitivity) plotted versus false positive rate (100-specificity) for different cutoff points for nucleosomes, CEA and CA 19-9.

## Discussion

Nuclear fragmentation as one of the morphologic features of apoptosis, results in a characteristic pattern of DNA complexed with histone proteins known as nucleosomes [18]. The measurement of these nucleosomes constitutes a feasible parameter for late stage of apoptosis [19]. Circulating nucleosomes can be quantified by real time PCR of the DNA. Moreover they can be estimated by stable immunologic assays that are particularly well suited for serial measurements.

CEA is a member of the immunoglobulin superfamily which was originally identified in human fetal colon and colorectal cancer. It is widely used as a tumor marker. However, little is known about its function except that it acts as a homotypic adhesion molecule that is implicated in cell aggregation [20]. It is over-expressed in numerous human cancers where it is present on the surface of cancer cells. It was reported that the over expression of CEA can protect cancer cells from apoptosis while a decrease in expression might lead to new approaches for management of cancer colon and other organs [21]. CEA is produced by more than 90% of colorectal cancers and contributes to the malignant characteristics of this type of cancer [22].

Although colorectal cancer screening is recommended for persons over 50 years and older its use is still low especially among older individuals [23].

An ideal tumor marker would be inexpensive screening that may help for early diagnosis in population at risk of cancer. Unfortunately currently available serological markers for colorectal carcinoma have not proven to be ideal [24].

The levels of serum nucleosomes show significant increase in patients as compared to control group (Table 1) with excellent discrimination between the two (area under curve equals 1 in table 2) that agrees with other published data [25]. There is an association of high nucleosomes levels with advanced stages of colorectal carcinoma. This may be due to a delayed clearance of nucleosomes from circulation where the bulky tumor tends to undergo peripheral apoptosis and central necrosis that in its turn would elicit local and systemic inflammatory responses and hence higher nucleosomes production [26].

The relationship between apoptosis and the degree of cell differentiation may play an important role in the susceptibility of the tumor cells to apoptosis [27]. An immature phenotype represents a block in the normal differentiation pathway [28]. Also, another study observed that the more the colon cells are stimulated to differentiate, the less likely they are to proliferate and hypothetically, the higher their rate of apoptosis [29].

After complete tumor resection of colorectal cancer, it was noticed that the level of nucleosomes increased in most patients rapidly reaching a maximum level during the first day. This was followed by a subsequent decrease, while the level was lower than in patients with postoperative or relapse therapy [30].

In the current study, the overall positive rates obtained from ROC curve (Figure 1) of circulating CEA and CA 19-9 (using the cutoff values of 3.56 ng/ml and 28 U/ml, respectively) were 56.2% and 36.4% (Table 2). These levels are higher than those reported by Zheng et al [24] who gave positive values of 29.2% and 25.2% for CEA and CA 19-9, their cutoff values of 5 ug/l and 31 Ku/l respectively. While the positive rates reported by Chan and Sell,[31], were 70% and 30% for CEA and CA 19-9, the cutoff values were of 3.5 ug/l and 37 Ku/l, respectively. CEA in the current study can provide a better discrimination between control subjects and patients than CA 19-9 (the areas under the ROC curve were 0.808 and 0.524 for CEA and CA 19-9, respectively).

Table (1) shows no significant difference in the levels of CA 19-9 in patients as compared to the control group while CEA shows a difference. Bhatnagar et al [32] noticed that in the well differentiated colorectal carcinoma there is more production of CEA/gram of total protein than in the poorly differentiated tumors in agreement with results obtained in the present study. On the other hand, it was observed that there is no significant correlation between levels of serum CA 19-9 and CEA [22] and the differentiation degree of the tumor.

## Conclusion

In conclusion, our study confirms that the levels of nucleosomes provide highly specific and sensitive apoptotic marker for colorectal carcinoma which should be applied on a large scale of cancers with respect to clinicopathological variables. It can be used - for diagnosis, screening, prognosis, and in therapy monitoring.

## List of abbreviations

CEA: Carcinoembryonic antigen; AFP: Alphafetoprotein; PSA: Prostate specific antigen; NCP: Nucleosome core particle; ECLISA: Electrochemi-luminescence immunoassay; ROC: Receiver operating characteristic.

## Competing interests

The authors declare that they have no competing interests.

## Authors' contributions

All authors contributed equally to this work and read and approved the final manuscript.

## References

[B1] Chan CC Fan CW Kuo YB Chen YH Chang PY Chen KT Hung RP Chan EC Multiple serological biomarkers for colorectal cancer detection Int J Cancer 2010 126 1683 1690 19795454 10.1002/ijc.24912

[B2] Zhao-Hut H Li-Hua L Fan Y Jin-Fu W Detection of apparent methylation in fecal DNA as a molecular screening tool for colorectal cancer and precancerous lesions World J Gastroenterol 2007 14 950 954 10.3748/wjg.v13.i6.950 PMC4065936 17352030

[B3] Ramos M Esteva M Cabeza E Campillo C Llobera J Aguilo A Relationship of diagnostic and therapeutic delay with survival in colorectal cancer: A review European journal of cancer 2007 43 2467 2478 10.1016/j.ejca.2007.08.023 17931854

[B4] Ahlquist DA Molecular Detection of Colorectal Neoplasia Gastroenterology 2010 138 2127 2139 10.1053/j.gastro.2010.01.055 20420950

[B5] Osaka A Hasegawa H Yamada Y Yanagihara K Hayashi T Mine M Aoyama M Sawada T A novel role of serum cytochrome c as a tumor marker in patients with operable cancer J Cancer Res Clin Oncol 2009 135 371 377 10.1007/s00432-008-0479-y 18825408 PMC12160134

[B6] Holdenrieder S Stieber P Boden MH Busch M Pawel JV Schalhom A Nagel D Seidel D Circulating nucleosomes in serum Ann N Y Acad Sci 2001 945 93 102 11708501 10.1111/j.1749-6632.2001.tb03869.x

[B7] Tan ML Ooi JP Ismail N Moad AIH Muhammad TST Programmed Cell Death Pathways and Current Antitumor Targets Pharmaceutical Research 2009 26 1547 1560 10.1007/s11095-009-9895-1 19407932

[B8] Hengartner MO The biochemistry of apoptosis Nature 2000 407 770 774 10.1038/35037710 11048727

[B9] Aleman MJ De Young MP Tress M Ketting P Perry GW Nara YR Inhibition of single minded 2 gene expression mediates tumor - selective apoptosis and differentiation in human colon cancer cells Proc Natl Acad Sci 2005 102 12765 12770 10.1073/pnas.0505484102 16129820 PMC1200285

[B10] Remacle-Bonnet M Garrouste F Baillat G Andre F Marvaldi J Pommier G Membrane rafts segregate pro-and anti-apoptotic insulin like growth factor- 1 receptor signaling in colon carcinoma cells stimulated by membranes of tumor necrosis factor superfamily Am J Pathol 2005 167 761 773 10.1016/S0002-9440(10)62049-4 16127155 PMC1698735

[B11] Brandt D Volkmann X Anstatt M Langer F Manns MP Schulze-Osthoff K Bantel H Serum biomarkers of cell death for monitoring therapy response of gastrointestinal carcinomas European journal of cancer 2010 4 1464 1473 10.1016/j.ejca.2010.01.037 20202824

[B12] Holdenrieder S Stieber P Apoptotic markers in cancer Clinical Biochemistry 2004 37 605 617 10.1016/j.clinbiochem.2004.05.003 15234242

[B13] Van Nieuwenhuijze AEM Van Lopik T Smeenk RJT Aarden LA Time between onset of apoptosis and release of nucleosomes from apoptotic cells: putative implications for systemic lupus erythematosus Ann Rheum Dis 2003 62 10 14 10.1136/ard.62.1.10 12480662 PMC1754285

[B14] Haier J Nicolson GL The role of tumor cell adhesion as an important factor in formation of distant colorectal metastasis Dis Colon Rectum 2001 44 876 884 10.1007/BF02234713 11391152

[B15] Flamen P Hoekstra OS Homans F Van Cutsem E Maes A Stroobants S Peeters M Penninckx F Filez L Bleichrodt RP Mortelmans L Unexplained rising carcinoembryonic antigen (CEA) in the postoperative surveillance of colorectal cancer: the utility of positron emission tomography (PET) European Journal of Cancer 2001 37 862 869 10.1016/S0959-8049(01)00049-1 11313174

[B16] Duffy MJ Van Dalen A Haglund C Hansson L Klapdof R Lamerz R Nilsson O Sturgron C Toplocan O Clinical utility of biochemical markers of CRC: European Group on Tumor marker (EGTM) guidelines Eur J Cancer 2003 39 718 727 10.1016/S0959-8049(02)00811-0 12651195

[B17] Astler VB Coller FA The prognostic significance of direct extension of carcinoma of the colon and rectum Ann Sur 1954 139 846 852 10.1097/00000658-195406000-00015 PMC1609522 13159135

[B18] Campbell PN Smith AD Biochemistry illustrated 2000 Edinburgh: Churchill Livingstone

[B19] Zeerleder S Zwart B Wuillemin WA Aarden LA Groeneveld ABJ Caliezit C Van Nieuwenhuijze AEM Van Mierlo GJ Eerenberg AJM Lammle B Hack CE Elevated nucleosome levels in systemic inflammation and sepsis Crit Care Med 2003 31 1947 1951 10.1097/01.CCM.0000074719.40109.95 12847387

[B20] Benchimol S Fuks A Jothy S Beauchemin N Shirota K Stanners CP Carcinoembryonic antigen, a human tumor marker, functions as intercellular molecule Cell 1989 57 327 334 10.1016/0092-8674(89)90970-7 2702691

[B21] Ueda K Iwahashi M Nakamori M Improvement of carcinoembryonic antigen-specific prodrug gene therapy for experimental.cancer colon Surgery 2003 133 309 317 10.1067/msy.2003.73 12660644

[B22] He Z Shi C Wen H Li F Wang B Wang J The potential of carcinoembryonic antigen, p53, Ki-67 and glutathione S-transferase-π as clinico-histopathological markers for colorectal cancer Journal of Biomedical Research 2010 24 51 57 10.1016/S1674-8301(10)60008-5 PMC3596535 23554611

[B23] Koroukian SM Xu F Dor A Woper GS Colorectal cancer screening in the elderly population: disparities by dual Medicare - Medicaid enrollment status Health Ser Res 2006 41 2136 2154 10.1111/j.1475-6773.2006.00585.x PMC1955310 17116113

[B24] Zheng CX Zhan WH Zhao JZ Zheng D Wang DP He YL Zheng ZQ The prognostic value of pre-operative serum levels of CEA, CA 19-9 and CA 72-4 in patients with colorectal cancer World J Gastroenterol 2001 7 431 434 11819806 10.3748/wjg.v7.i3.431 PMC4688738

[B25] Holdenrieder S Nagel D Schalhorn A Heinemann V Wilkowski R Von Pawel J Raith H Feldmann K Kremer AE Muller S Geiger S Hamann GF Seidel D Stieber P Clinical Relevance of Circulating Nucleosomes in Cancer Ann N Y Acad Sci 2008 1137 180 189 10.1196/annals.1448.012 18837945

[B26] Wigmore SJ McMahon AJ Sturgeon CM Fearon CH Acute-phase protein response, survival and tumor recurrence in patients with colorectal cancer Br J Surg 2001 88 255 260 10.1046/j.1365-2168.2001.01669.x 11167877

[B27] Shoji Y Saegusa M Tanako Y Ohbu M Okayasu I Correlation of apoptosis with tumor cell differentiation, progression and HPV infection in cervical carcinoma J Clin Pathol 1996 49 134 138 10.1136/jcp.49.2.134 8655679 PMC500346

[B28] Beere HM Hickman JA Differentiation: A suitable strategy for cancer chemotherapy Anticancer drug Des 1993 8 299 322 8240658

[B29] Jenab M Thompson LU Phytic acid in wheat bran affects colon morphology, cell differentiation and apoptosis Carcinogenesis 2000 21 1547 1552 10.1093/carcin/21.8.1547 10910957

[B30] Andreas K Stefan H Petra S Ralf W Dorothea N Dietrich S Nucleosomes in colorectal cancer patients during radiochemotherapy Tumor Biology 2006 27 235 242 10.1159/000094694 16864976

[B31] Chan DW Sell S Burtis CA, Ashwood ER Tumor markers Tietz Textbook of Clinical Chemistry 1999 Philadelphia: W B Saunders Company 722 724

[B32] Bhatnagar J Tewari H Bhatnagar M Austin GE Comparison of carcinoembryonic antigen in tissue and serum with grade and stage of colon cancer Anticancer Res 1999 19 2181 2187 10472328

